# Ponatinib-induced eruptive squamous cell carcinomas

**DOI:** 10.1016/j.jdcr.2025.04.032

**Published:** 2025-05-21

**Authors:** Gabrielle Moore, Alexander Gross

**Affiliations:** a Edward Via College of Osteopathic Medicine, Blacksburg, Virginia; b Department of Dermatology, Emory University School of Medicine, Atlanta and Cumming, Georgia

**Keywords:** ponatinib, squamous cell carcinoma, tyrosine-kinase inhibitor

## Introduction

Philadelphia chromosome (Ph), t(9;22), is the most common cytogenetic abnormality in patients diagnosed with acute lymphoblastic leukemia. This is the result of a fusion between the ABL1 gene on chromosome 9 and the BCR gene on chromosome 22, creating an oncoprotein.^1^ To target this fusion, ponatinib, a BCR:ABL1 tyrosine kinase inhibitor (TKI) with high potency against Ph-positive leukemias, was created.^2^ Thus, ponatinib was approved to treat acute lymphoblastic leukemia and has shown high efficacy rates.^1^ Although ponatinib is effective with a 73% 5-year overall survival, there are some notable adverse events of the drug. The main concern for adverse events of ponatinib in the literature is the associated cardiotoxicity. There has also been evidence of severe side effects of ponatinib, including pancreatitis, atrial fibrillation, pneumonia, and angina pectoris.^3^ However, 1 rare side effect that has seldom been noted in literature is eruptive well-differentiated squamous cell carcinomas (SCCs). This adverse effect has only been previously described with the use of a similar TKI, nilotinib, and has yet to be documented with ponatinib.^4^^,^^5^ Here, we discuss a 78-year-old Caucasian female who presented with a sudden onset of multiple scaly lesions on the bilateral lower extremities after beginning treatment with ponatinib 1 month prior. One punch biopsy and 2 shave biopsies were performed, revealing well-differentiated SCCs. The sudden onset of this severe adverse effect requires prompt monitoring by the medical oncologists and dermatologists involved in the patient’s care to ensure timely intervention. Likewise, it would be beneficial for the physician to counsel the patient on this potential adverse effect before initiating the medication.

## Case presentation

**Fig 1:**
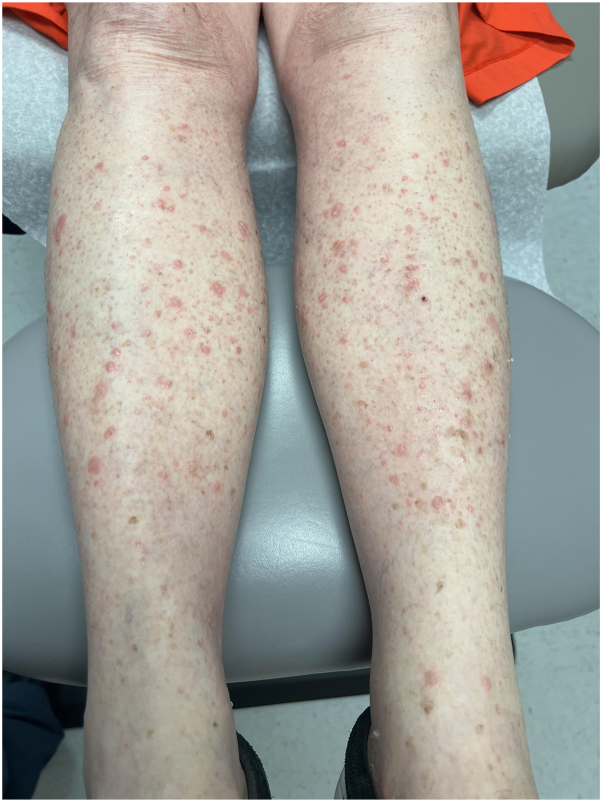
Clinical image on presentation showed eruptive squamous cell carcinomas on the bilateral lower extremities.

A 78-year-old female with an underlying diagnosis of Ph-positive (Ph+) acute lymphoblastic leukemia presented with a sudden onset of multiple scaly lesions on the bilateral lower extremities 1 month after beginning treatment with ponatinib (shown in Fig 1). The patient reported mild pain associated with the lesions. The patient denied any associated constitutional symptoms or history of any similar skin lesions. Medications included acyclovir 400 mg/day, amiodarone 200 mg/day, cholecalciferol 200 mcg/day, diltiazem 180 mg/day, famotidine 20 mg/day, fluconazole 200 mg/day, methocarbamol 750 mg/day, torsemide 20 mg/day, tramadol 50 mg/day, rivaroxaban 20 mg/day, and ponatinib 30 mg/day. Punch biopsies from the lesions revealed keratinocyte atypia with the epidermis extending into the underlying dermis with an invasive pattern of growth. The neoplastic cells display pleomorphic features with nuclear hyperchromasia. A diagnosis of well-differentiated SCC was confirmed. The treatment plan included acitretin 25 mg daily with discontinuation of ponatinib.

## Discussion

**Fig 2:**
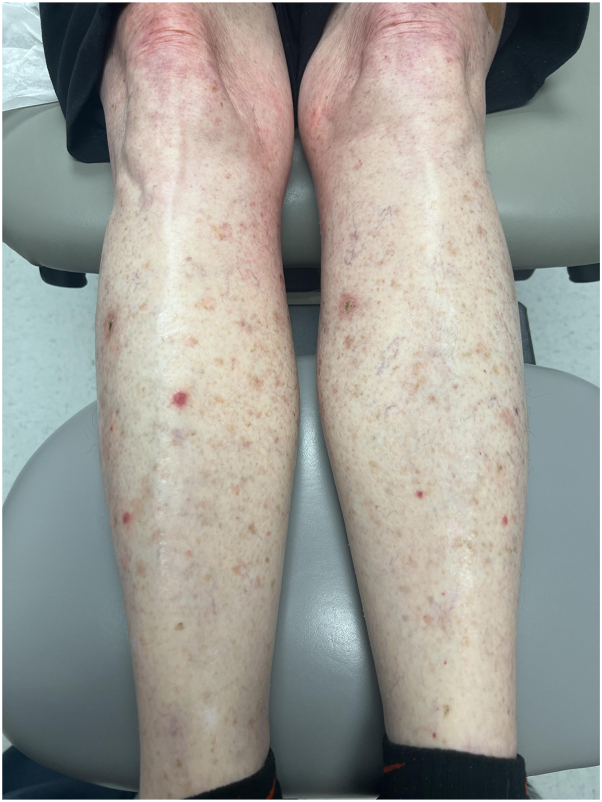
Clinical image of healed eruptive squamous cell carcinomas on the bilateral lower extremities after 2 months of acitretin treatment.

**Fig 3:**
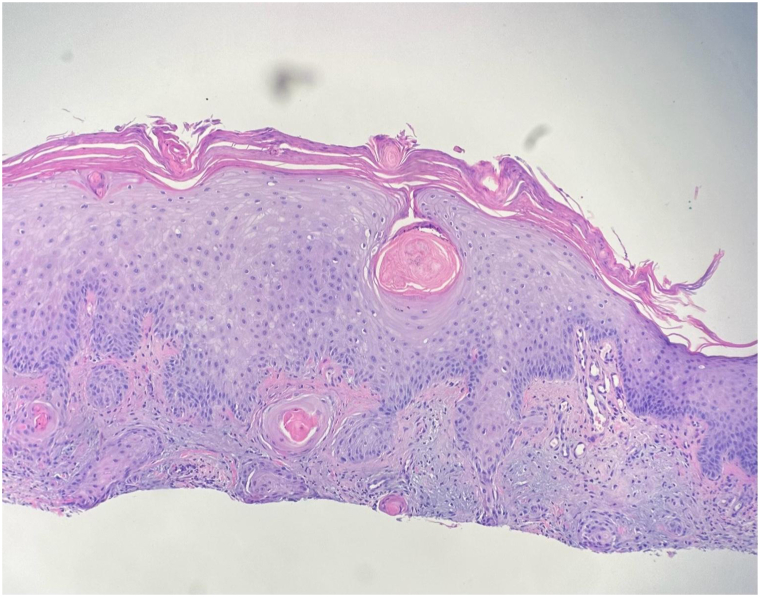
Hematoxylin and eosin stain (4×), keratinocyte atypia with the epidermis extending into the underlying dermis with an invasive pattern of growth. The neoplastic cells display pleomorphic features with nuclear hyperchromasia.

As mentioned previously, this adverse effect of eruptive SCCs has been noted with another TKI, nilotinib. When studied, it was found that ponatinib also affects the BCR-ABL-dependent mitogen-activated protein kinase pathway similar to nilotinib. In the presence of preexisting mutations related to UV radiation, the unchecked activation can result in the development of the SCCs that we saw in this patient.^4^^,^^6^ Although TKIs can result in life-threatening severe cutaneous adverse reactions, the BCR-ABL protein TKIs may cause the eruption of SCCs that are not expected to metastize.^7^ Due to the rapid onset of SCCs in this patient, relative to the use of ponatinib and documented side effects of the drug, ponatinib is suspected to be the trigger of these eruptions. Pertinent negatives for consideration of cause included xeroderma pigmentosa, oculocutaneous albinism, and human papillomavirus-driven SCCs. One other mimicker that should be considered is hypertrophic lichen planus (HLP). HLP shares many clinical and histologic characteristics with SCC, and HLP can undergo malignant transformation to develop SCC. In this patient, the eruptive SCCs were found on the shins, similar to where HLP would present as well. However, upon review of the histopathology (shown in Fig 3), the sections show keratinocyte atypia with the epidermis extending into the underlying dermis with an invasive pattern of growth. The neoplastic cells display pleomorphic features with nuclear hyperchromasia. Thus, the findings are consistent with invasive, well-differentiated SCC. Given the clinical presentation, this represents eruptive SCC secondary to treatment with a TKI, rather than HLP. Of note, this is an important clinical distinction.^8^ In the prior nilotinib case, the patient was treated with acitretin with positive results while the patient continued with their TKI treatment.^4^ In this case, the TKI treatment was discontinued, per decision of the oncologist to halt further SCC formation, and acitretin 25 mg daily was used to treat the eruption that resolved over 30 days. Acitretin is a second-generation retinoid that exhibits antitumor activity against SCCs, possibly by modulating gene expression and suppressing epithelial carcinogenesis within the skin.^9^ While there is high efficacy of acitretin, acitretin monotherapy is also documented to exhibit low-grade side effects; thus, the use of acitretin is safe for patients, even with immunosuppression.^10^ In this case, the patient did not suffer from any adverse side effects and was effectively treated with acitretin monotherapy (shown in Fig 2). This case is important to note as one of the first accounts of ponatinib-induced eruptive SCCs with effective treatment by acitretin. Additionally, awareness by medical oncologists and dermatologists of this rare adverse effect from ponatinib use is imperative to recognize diffuse eruptive SCCs and avoid excessive use of surgical interventions.

## Conflicts of interest

None disclosed.
